# Scurvy in an Adolescent: A Case Report

**DOI:** 10.7759/cureus.58782

**Published:** 2024-04-22

**Authors:** Tony Du, Abhay Kulkarni

**Affiliations:** 1 Pediatric Emergency Medicine, Baylor College of Medicine, Houston, USA

**Keywords:** scurvy, vitamin c, diet, anorexia nervosa, corkscrew hairs, perifollicular hemorrhage, ascorbic acid

## Abstract

Scurvy is a rare condition characterized by a deficiency in dietary vitamin C. Historically a disease taught in the context of long ocean voyages with limited vitamin intake, it is now rare in developed nations. The classical physical exam findings include gingival bleeding, perifollicular hemorrhages, and corkscrew hairs. We discuss the case of a 15-year-old female with scurvy whose initial presentation suggested more common diagnoses seen in the emergency department setting. Her course was complicated by a prior history of anorexia nervosa and a restrictive diet that lacked necessary vitamins. Once the patient’s dietary habits were identified, a detailed physical exam revealed the characteristic findings. She was subsequently discharged with oral vitamin C supplements and was scheduled for outpatient follow-up to monitor symptoms.

## Introduction

Scurvy is a disease resulting from a dietary deficiency of vitamin C, one historically common among sailors on long voyages with limited vitamin intake [1]. It is now rarely seen in developed countries where a balanced diet is the norm, but the majority of scurvy cases remain in high-risk populations such as alcoholics, malnourished individuals, and those with limited access to nutritional foods [2]. This deficiency impairs collagen synthesis, which is crucial for providing structural support to blood vessels and hair follicles. It classically presents with symptoms such as anemia, gingival bleeding, arthralgias, perifollicular hemorrhages, and corkscrew hairs [3]. While the diagnosis of scurvy can be made clinically, it is confirmed by laboratory tests indicating low levels of serum ascorbic acid. Treatment involves vitamin C supplementation and a diet rich in fruits and vegetables [4].

## Case presentation

A 15-year-old female with a past medical history of anorexia nervosa presented to the emergency department with a rash on both legs that had been progressive and spreading caudally over the past seven days, denying any pain or pruritus. She also reported gingival bleeding while brushing her teeth, a “bump” on her left knee, menorrhagia, and more fatigue when walking. She had no fever, chills, or abdominal pain and was not taking any medications. Paternal family history revealed a history of unspecified malignancies.

**Figure 1:**
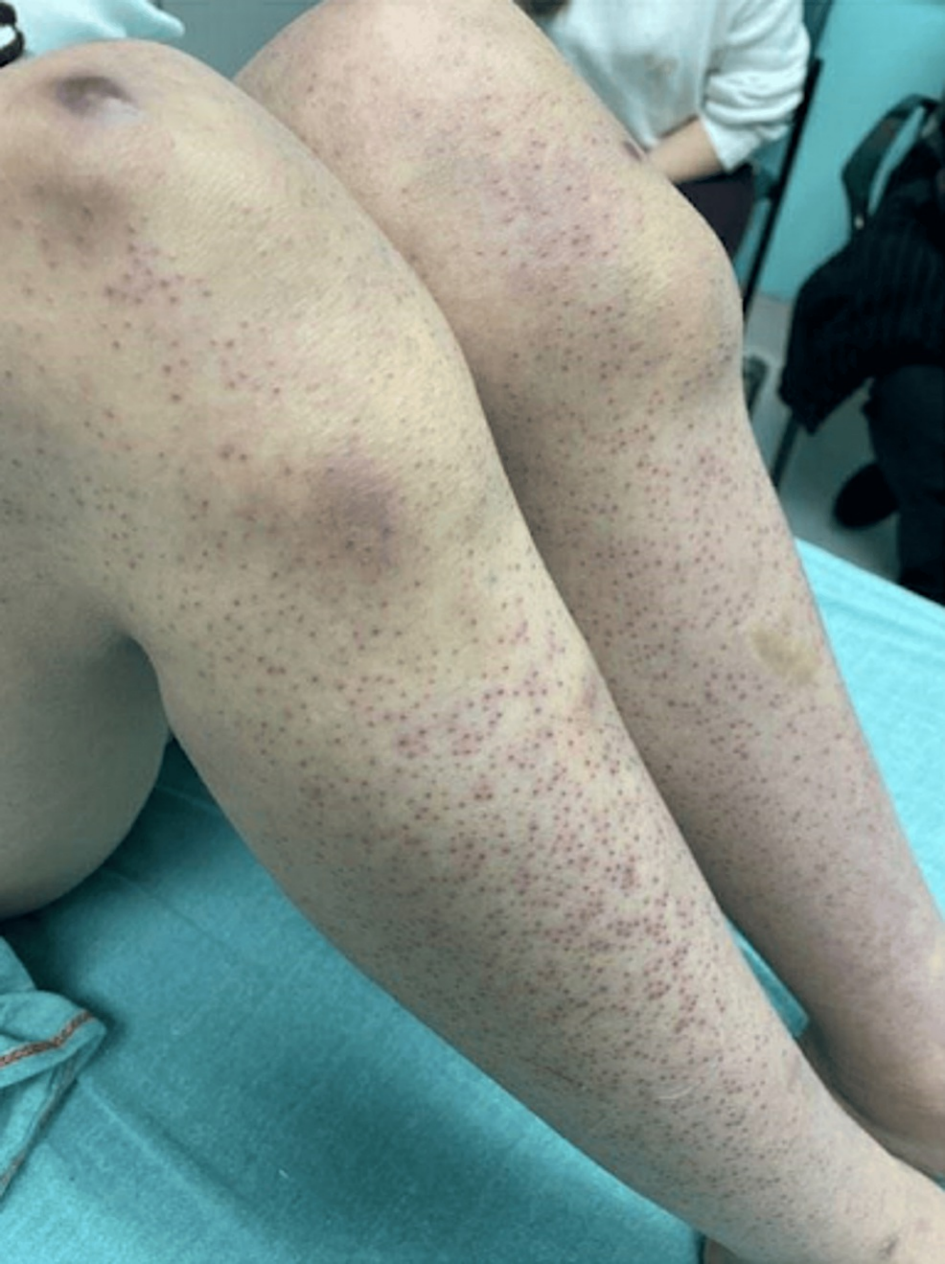
Subcutaneous fluid collection to the left lower extremity.

**Figure 2:**
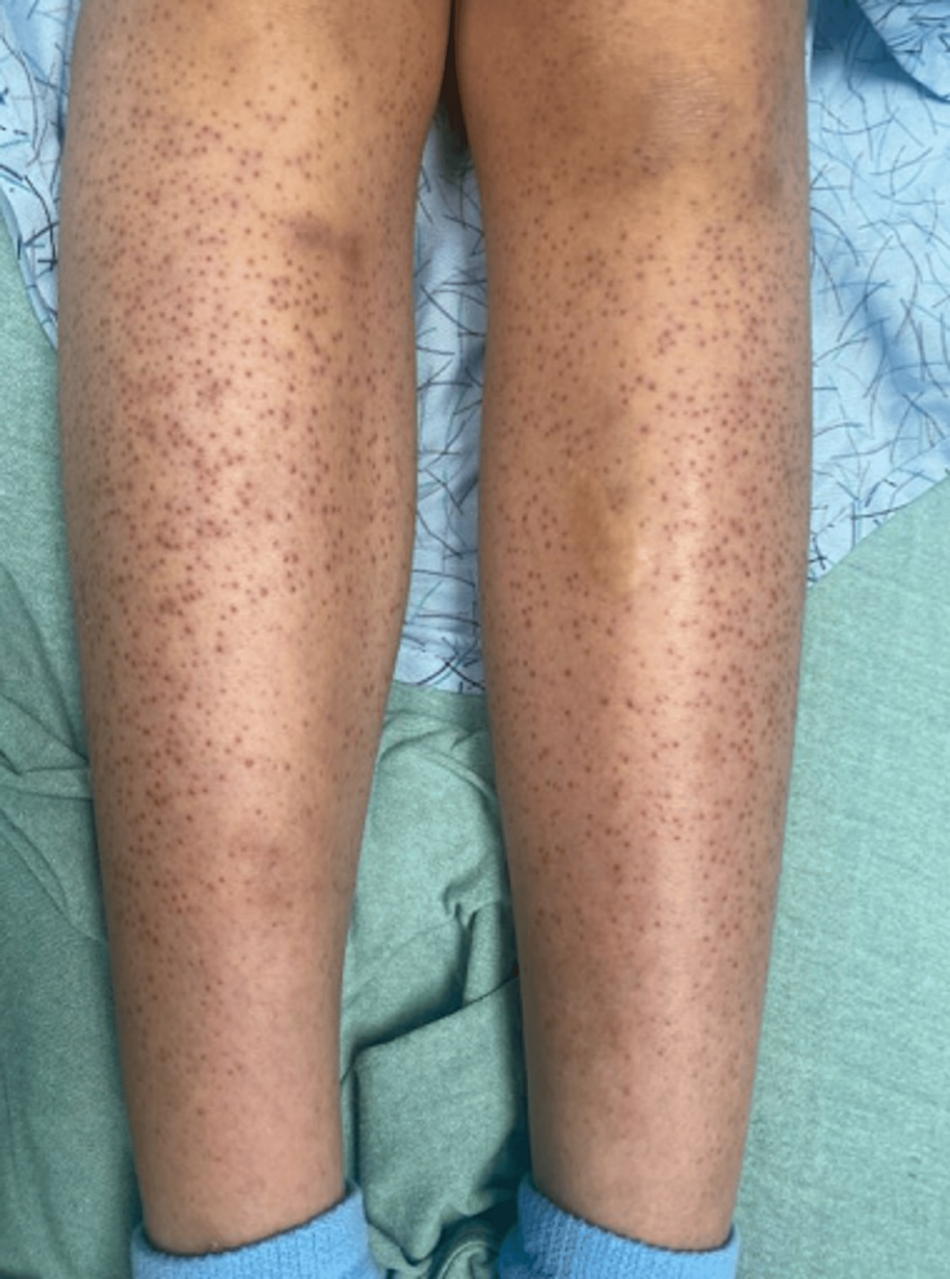
Non-blanching, perifollicular papules across the bilateral lower extremities.

On examination, the patient was tachycardic with mild swelling on her left cheek, scant cervical lymphadenopathy, and a soft, subcutaneous fluid collection to the left knee (Figure 1). There were non-blanching, perifollicular papules across the bilateral lower and upper extremities (Figure 2). Our differential diagnoses included autoimmune disorders, malignancy, coagulopathic disorders, Henoch-Schönlein purpura, and bacterial infection, among others.

The initial workup involved obtaining a complete blood count (CBC), basic metabolic panel (BMP), blood cultures, urinalysis, coagulation panels, and plain films of the chest and lower extremities. CBC revealed no abnormalities, notably with a normal white blood count and hemoglobin, aside from a minor increase in eosinophils. BMP resulted in no significant metabolic derangements. Coagulation studies revealed an increase in D-dimer. Blood cultures, urinalysis, and plain films were all within normal limits. To further evaluate the dermatologic abnormalities, dermatology was consulted to examine the papules.

The possibility of nutritional deficiencies was considered, and a detailed dietary history was obtained. Discussion with the patient’s guardians revealed that she had a very selective diet, limited to mostly peanut butter and jelly sandwiches and macaroni and cheese, noting that she only drinks water and rarely eats fruits or vegetables. Supported by the history of limited food intake and history of anorexia nervosa, our differential diagnoses broadened to include vitamin C deficiency.

**Figure 3:**
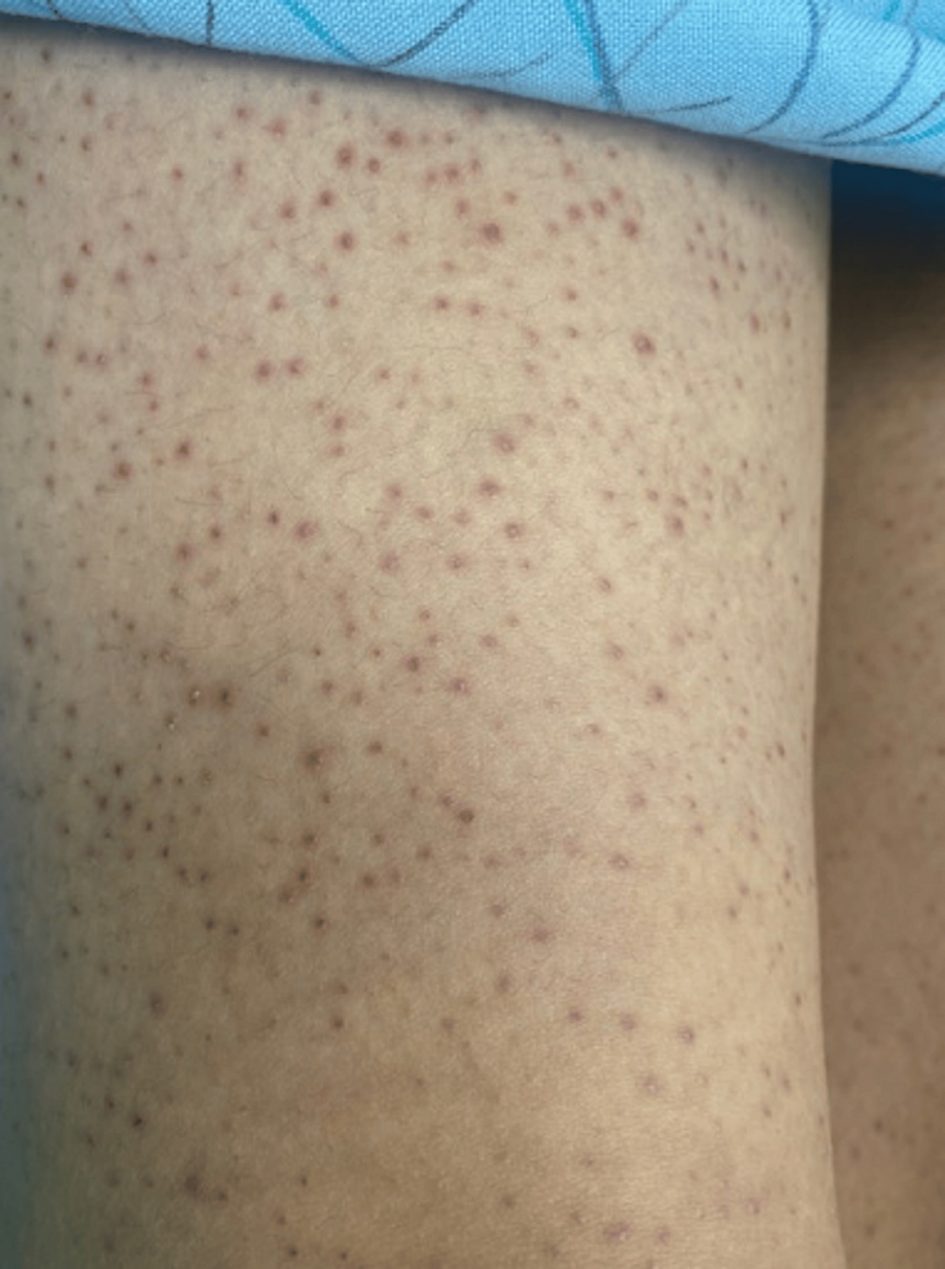
Faint corkscrew hairs.

**Figure 4:**
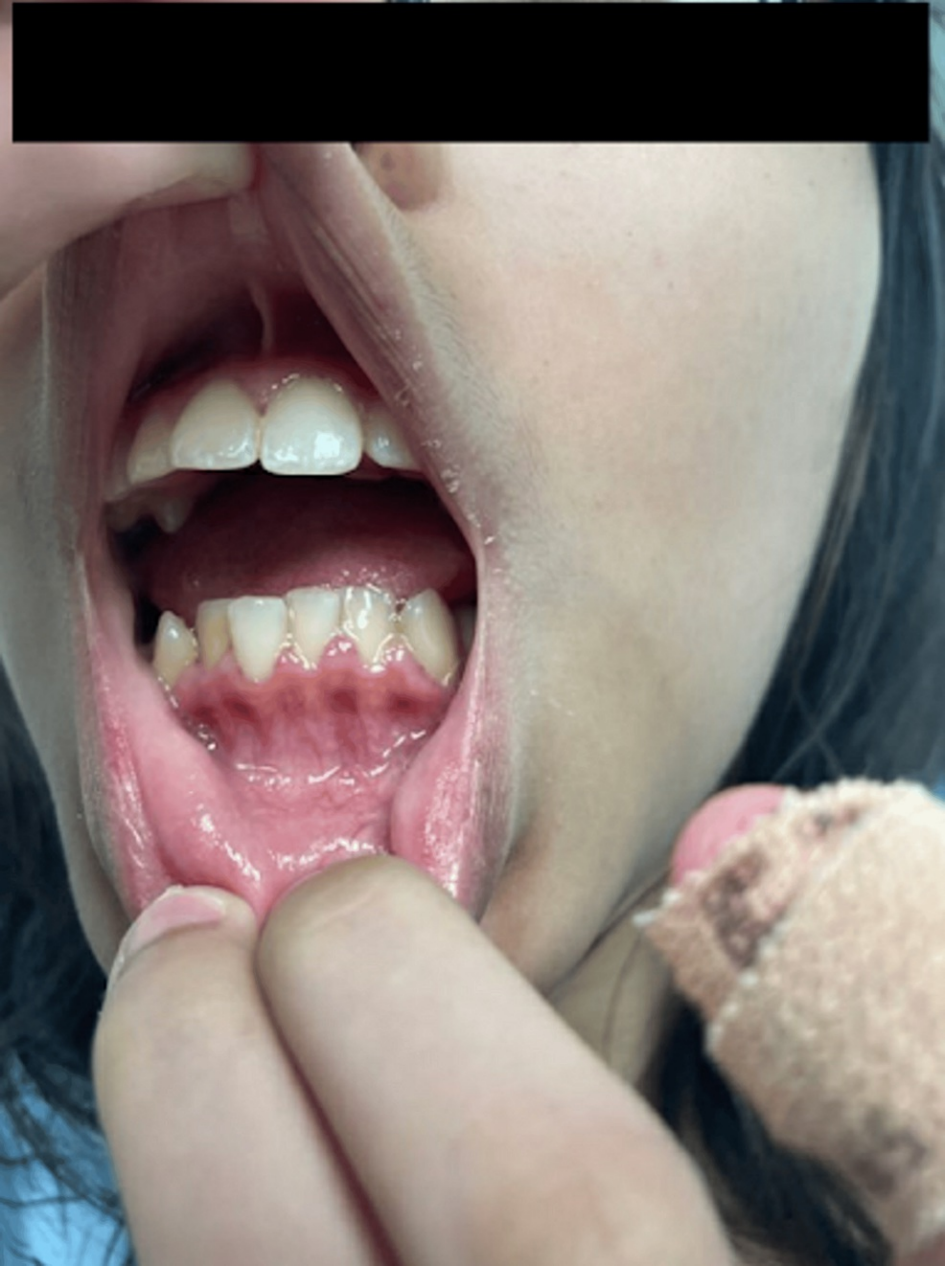
Gingival irritation along the lower gingiva.

A thorough skin examination was subsequently performed, revealing perifollicular hemorrhages and faint corkscrew hairs involving the bilateral lower extremities (Figure 3). Oral examination also revealed gingival irritation along the lower gum line (Figure 4). A clinical diagnosis of scurvy was made, and the patient was discharged with oral vitamin C supplements. The patient’s serum vitamin C level was measured four days following discharge and was found to be <5 µmol/L (reference range: 24-114 µmol/L). Vitamin C levels between 11-23 µmol/L indicate a moderate risk for a deficiency, and levels <11 µmol/L indicate severe deficiency. The patient is currently scheduled to follow up with outpatient care to monitor for improvement of her symptoms.

## Discussion

Scurvy is a well-documented nutritional deficiency that historically affected sailors with limited access to vitamin C-rich foods [1]. While rare in developed countries, 5.9% of the population in the United States is still affected by vitamin C deficiency, which is a decrease from the previously recorded 7.1% [5,6]. The importance of a healthy diet in preventing scurvy cannot be overstated since pediatric scurvy is still prevalent among children with limited diets and aversions to vitamin C-rich fruits and vegetables [7]. This is compounded by individuals with a history of eating disorders, particularly anorexia nervosa, where an aversion to consuming nutritious foods may exacerbate any underlying nutritional deficiencies.

Vitamin C is essential in collagen synthesis, acting as a coenzyme and providing structural support to blood vessels and hair follicles [8]. A deficiency in vitamin C hinders collagen production, leading to various clinical manifestations. In cases of vitamin C deficiency, blood vessels exhibit increased fragility and susceptibility to rupture, resulting in characteristic gingival bleeding and perifollicular hemorrhages. Hair follicles may also become weakened and curl around keratinous material as it protrudes from the skin, leading to its distinctive corkscrew appearance [3]. In our case, these findings were all apparent.

Scurvy presents with a range of dermatologic, hematologic, and musculoskeletal changes, making the differential diagnosis broad and including malignancy, vasculitides, coagulation disorders, and rheumatologic diseases [4,9,10]. As a result, scurvy may be easily overlooked, particularly in an emergency department setting. Thus, obtaining a detailed nutritional history is crucial, particularly in at-risk populations. In patients with previously treated eating disorders, particularly anorexia nervosa, careful monitoring of dietary intake (and inclusion into patient history) in these groups should be maintained to avoid any sequelae of nutritional deficiencies. The persistence of nutritional deficiencies following treatment of anorexia nervosa has been well-documented, with some studies recommending vitamin supplementation well after weight recovery [11].

The complications linked to scurvy pose a significant risk to life. Untreated scurvy in its advanced stages can lead to spontaneous bleeding, widespread edema, and, ultimately, death due to complications arising from internal bleeding or infection [3]. The treatment typically involves vitamin C supplementation and a diet rich in fruits and vegetables. Children commonly receive daily doses of 100-300 mg of vitamin C, whereas adults are usually prescribed 500-1000 mg per day for one month or until all clinical signs and symptoms have completely resolved. Patients typically respond well to this therapy, and improvement within days to weeks is usually seen [4].

## Conclusions

Nutritional deficiencies, notably vitamin C deficiency, should be considered in the differential diagnosis of patients exhibiting dermatologic and hematologic changes, especially among those with a history of psychiatric disorders or unconventional dietary habits. In our case, the patient’s gingival bleeding, perifollicular hemorrhages, corkscrew hairs, and limited diet were consistent with scurvy. Maintaining a high index of suspicion is particularly crucial, particularly in the emergency department, where nutritional deficiencies may be easily overlooked. Early recognition and management of vitamin C deficiency are vital for preventing further complications and improving patient outcomes.
